# Supplementary material for: The causal role of circulating inflammatory markers in osteoporosis: a bidirectional Mendelian randomized study
Source: Front Immunol. 2024 Jul 18;15:1412298. doi: 10.3389/fimmu.2024.1412298 (PMC11291241; doi:10.3389/fimmu.2024.1412298)
Supplement: Supplementary file 1 [file DataSheet_1.zip › supplementary material/CIM-OSTEOPOROSIS /GCST90274840.csv_forest.pdf]

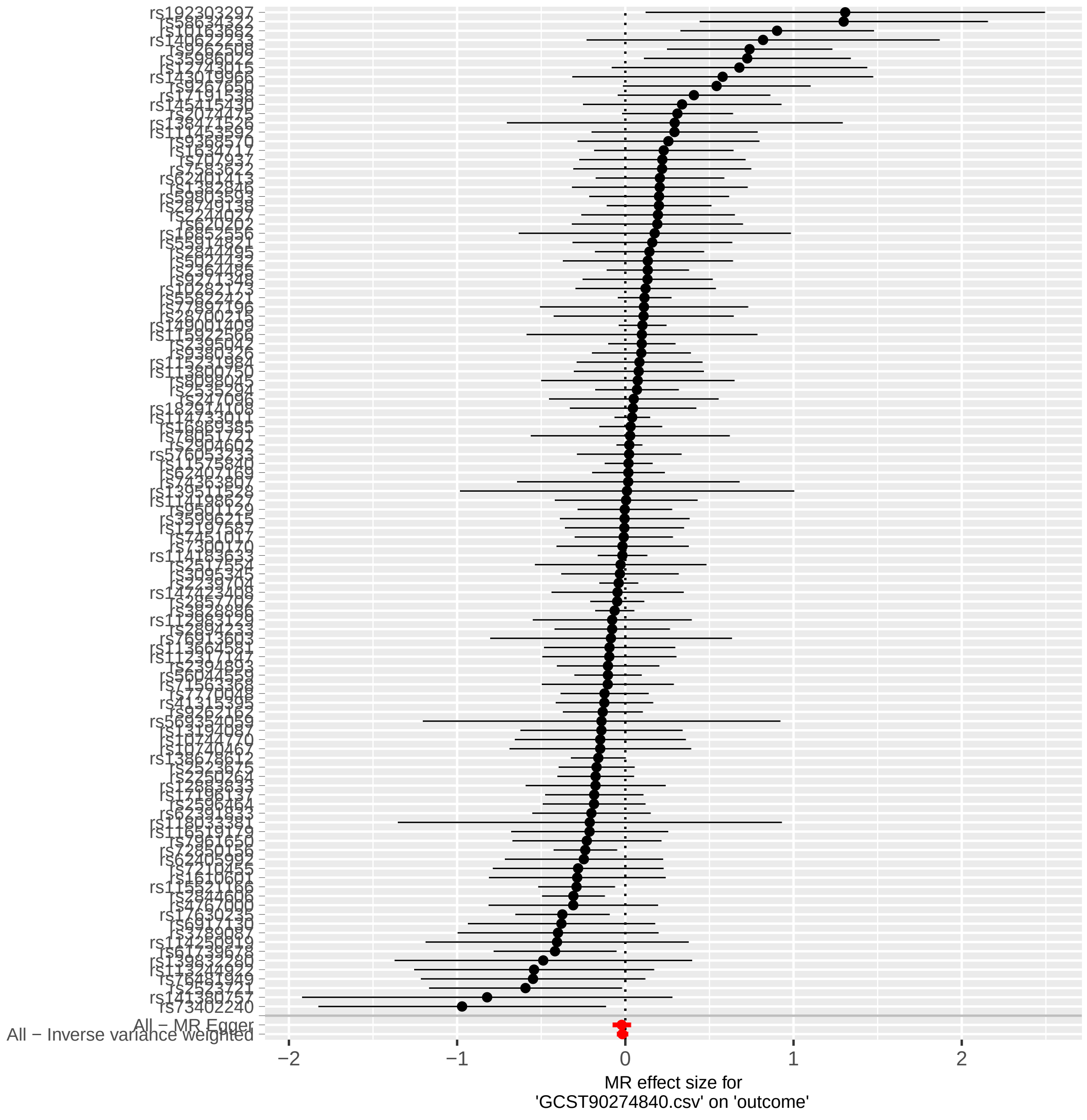
rs192303297
rs145415430
rs7583622
rs62401413
rs149001409
rs115922566
rs247096
rs182914108
rs11575840
rs62407169
rs139511528
rs7300170
rs114183633
rs112983129
rs76913603
rs7770048
rs41315395
rs138678612
rs2523675
rs2250264
rs7961650
rs62405992
rs17630235
rs6917130
rs3789087
rs114250919
rs76481949
rs2523721
rs141380757
rs73402240
All − MR Egger
All − Inverse variance weighted
−2
−1
0
1
2
MR effect size for
'GCST90274840.csv' on 'outcome'
